# The impact of extraction protocol on the chemical profile of cannabis extracts from a single cultivar

**DOI:** 10.1038/s41598-021-01378-0

**Published:** 2021-11-08

**Authors:** Janina K. Bowen, Jacqueline M. Chaparro, Alexander M. McCorkle, Edward Palumbo, Jessica E. Prenni

**Affiliations:** 1 Department of Horticulture and Landscape Architecture, Colorado State University, Fort Collins, CO USA; 2 Charlotte’s Web Inc., 1600 Pearl St., Boulder, CO 80302 USA

**Keywords:** Analytical chemistry, Mass spectrometry, Plant sciences

## Abstract

The last two decades have seen a dramatic shift in cannabis legislation around the world. Cannabis products are now widely available and commercial production and use of phytocannabinoid products is rapidly growing. However, this growth is outpacing the research needed to elucidate the therapeutic efficacy of the myriad of chemical compounds found primarily in the flower of the female cannabis plant. This lack of research and corresponding regulation has resulted in processing methods, products, and terminology that are variable and confusing for consumers. Importantly, the impact of processing methods on the resulting chemical profile of full spectrum cannabis extracts is not well understood. As a first step in addressing this knowledge gap we have utilized a combination of analytical approaches to characterize the broad chemical composition of a single cannabis cultivar that was processed using previously optimized and commonly used commercial extraction protocols including alcoholic solvents and super critical carbon dioxide. Significant variation in the bioactive chemical profile was observed in the extracts resulting from the different protocols demonstrating the need for further research regarding the influence of processing on therapeutic efficacy as well as the importance of labeling in the marketing of multi-component cannabis products.

## Introduction

*Cannabis sativa* L. is a pharmacologically important annual plant that produces bioactive phytocannabinoids and other secondary metabolites that have demonstrated therapeutic potential for a wide variety of human health conditions^1–5^. *Cannabis sativa* L. can be broadly divided into three categories based on genomic diversity and chemical composition^6^. Specifically, based on the analysis of 340 cannabis varieties including grain hemp, fiber hemp, CBD hemp, marijuana, and feral populations, the distinct groups were described as (1) fiber/grain hemp with low cannabinoid content, (2) cannabis with narrow leaflets (colloquially described as sativa) and high cannabinoid content (CBD hemp and marijuana), and (3) cannabis with broad leaflets (colloquially described as indica) and high cannabinoid content (CBD hemp and marijuana).

Δ^9^THC and CBD are the most extensively studied *Cannabis sativa* L derived phytocannabinoids and are the only compounds currently available by prescription in the United States^7^. In addition to these two major neutral phytocannabinoids, acidic (Δ^9^THCA, CBDA, CBGA, CBCA), minor (CBG, CBN, CBC), and varinic (Δ^9^THCV, CBDV, CBGV) phytocannabinoids have also exhibited promising in vitro and in vivo results for treatment of various human health conditions^4^. For example, as reviewed by Franco et. al. (2020), there is preliminary evidence that these understudied bioactive compounds have anti-inflammatory, anti-microbial, anti-proliferative, anti-convulsive and neuroprotective properties. Furthermore, these minor phytocannabinoids are emerging as potential treatment strategies for anxiety, nausea, diabetes, acne, metabolic syndrome, obesity, pain, colorectal cancer, breast cancer and more. Finally, in addition to phytocannabinoid compounds, there are a multitude of other bioactive compounds found in cannabis including terpenes and terpenoids^8–12^, flavonoids^13,14^, bibenzyls^15^, stilbenoids^16,17^, and hydroxycinnamic acids^18,19^.

There is a growing body of work exploring cannabis polypharmacy in terms of potential synergistic effects, commonly referred to as the entourage effect, that may contribute to or modulate the therapeutic properties of cannabis extracts. Synergistic effects have been proposed in research exploring combinations of phytocannabinoids^20,21^ as well as other bioactive secondary metabolites such as terpenes and/or terpenoids^22,23^. This has also been shown with human endocannabinoids in vitro, though in vivo studies are notably lacking. For example, it has been demonstrated that the endocannabinoid 2-arachidonoyl-glycerol shows enhanced activity in the presence of 2-acyl-glycerol esters, which alone are inactive^24^. This effect has also been noted for organisms other than cannabis. Combining multiple terpenes from a tropical Amazonian plant was demonstrated to have a synergistic effect that was more toxic to a parasite than the terpenes alone^12^ and combining multiple terpenes was more effective at inhibiting growth of a protozoa than the terpenes alone^25^. Conversely, there is also some evidence suggesting that cannabis polypharmacy could results in negative interactions or potential toxicity^26^.

Many distillate and isolate products are readily available to consumers including those containing CBD, CBDV, CBC, CBG, CBGA, CBN, Δ^8^THC, Δ^9^THC, and THCV. All but Δ^9^THC products can be purchased online and shipped anywhere in the United States. There has also been a surge in marketing of so called ‘full spectrum’ products which capitalize on the idea of the synergistic entourage effect. However, because these products are not regulated by the FDA as dietary supplements there is not a clear definition of what denotes a high-quality cannabis product, and phrases such as ‘whole plant’, ‘full spectrum’, and ‘broad spectrum’ further muddy the waters for consumers.

The composition of a commercial cannabis extract will in large part be determined by the genetics of the starting plant material^27^. While previous work has evaluated extraction parameters with the goal of optimizing recovery of the major phytocannabinoids^28–30^, the impact of processing methodology on the comprehensive composition of full spectrum extracts is not well understood. Commonly utilized commercial extraction approaches include the use of alcoholic solvents (e.g. ethanol and isopropanol) to more advanced technologies using supercritical CO_2_. Solvent extraction represents the lowest cost option; however, this method runs the risk of leaving behind trace organic solvent contamination. This is more of a concern with hydrocarbon solvents such as methanol, acetone and butane which are toxic for human consumption. Extraction with supercritical CO_2_ requires investment in specialized equipment but has multiple advantages including “tunability” by modifying temperature and pressure conditions for more precise extraction, the potential reuse of CO_2_^31^, and the lack of any residual solvents.

Given the lack of research into the therapeutic effects of phytocannabinoids and other bioactive secondary metabolites in humans, coupled with the potential for synergistic effects and variability in commercial processing methods, there is a critical need for additional research to characterize the numerous chemical compounds found in cannabis extracts and how this chemical profile is impacted by production choices. As a first step, we have conducted a comprehensive qualitative chemical analysis of cannabis extracts from a single high-CBD cultivar generated using three previously optimized commercial extraction protocols. The results of this study lay the groundwork for evaluation of the impact of processing method on chemical variation in full spectrum consumer products and represent an important step towards enabling industry standardization.

## Results and discussion

Cannabis extracts were generated from a single proprietary cultivar using previously optimized and commonly used commercial extraction procedures including alcoholic extraction with ethanol and isopropanol and extraction with super critical fluid CO_2_. For the latter, two fractions were generated for analysis, S1 and S2, corresponding to different pressure settings during the extraction. Each extract was analyzed using a combination of complementary analytical approaches to ensure broad chemical coverage. Overall, 41 compounds were detected and annotated by gas chromatography mass spectrometry (GC–MS) using a non-targeted profiling approach, 15 phytocannabinoids were evaluated by ultra-performance liquid chromatography tandem mass spectrometry (UPLC-MS/MS) using a qualitative targeted assay, and 24 elements were quantified by inductively coupled plasma mass spectrometry (ICP-MS).

**Figure 1:**
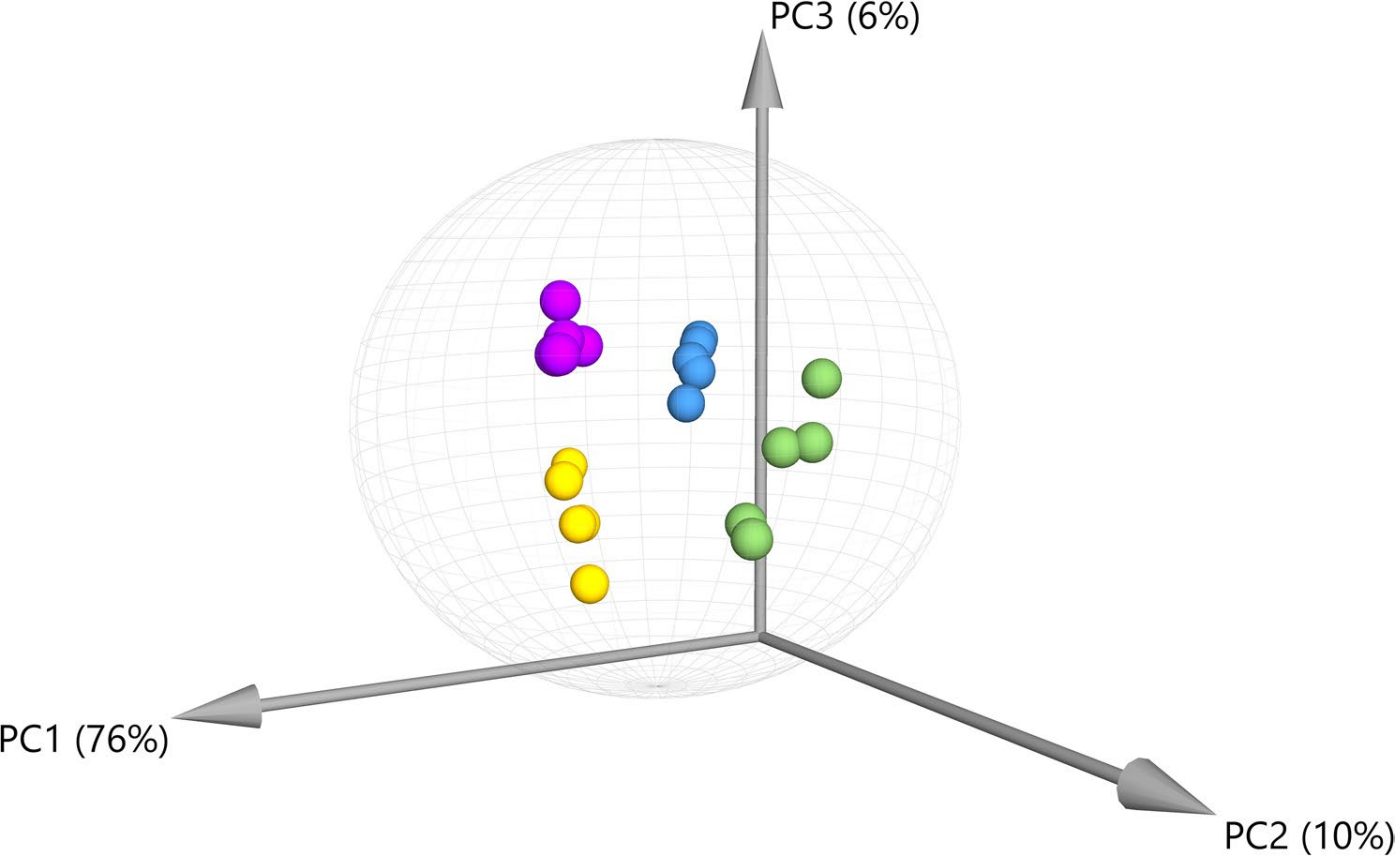
Principal Component Analysis (PCA) for compounds detected by GC–MS demonstrating the overall variation in chemical profiles generated from each extraction. The majority of variation (76%) was observed in the first principal component (PC1). Green = ethanol extract; Blue = isopropanol extract; Purple and Yellow = super critical CO_2_ fractions S1 and S2, respectively. Ellipse indicates 95% confidence using Hotelling’s T2.

**Figure 2:**
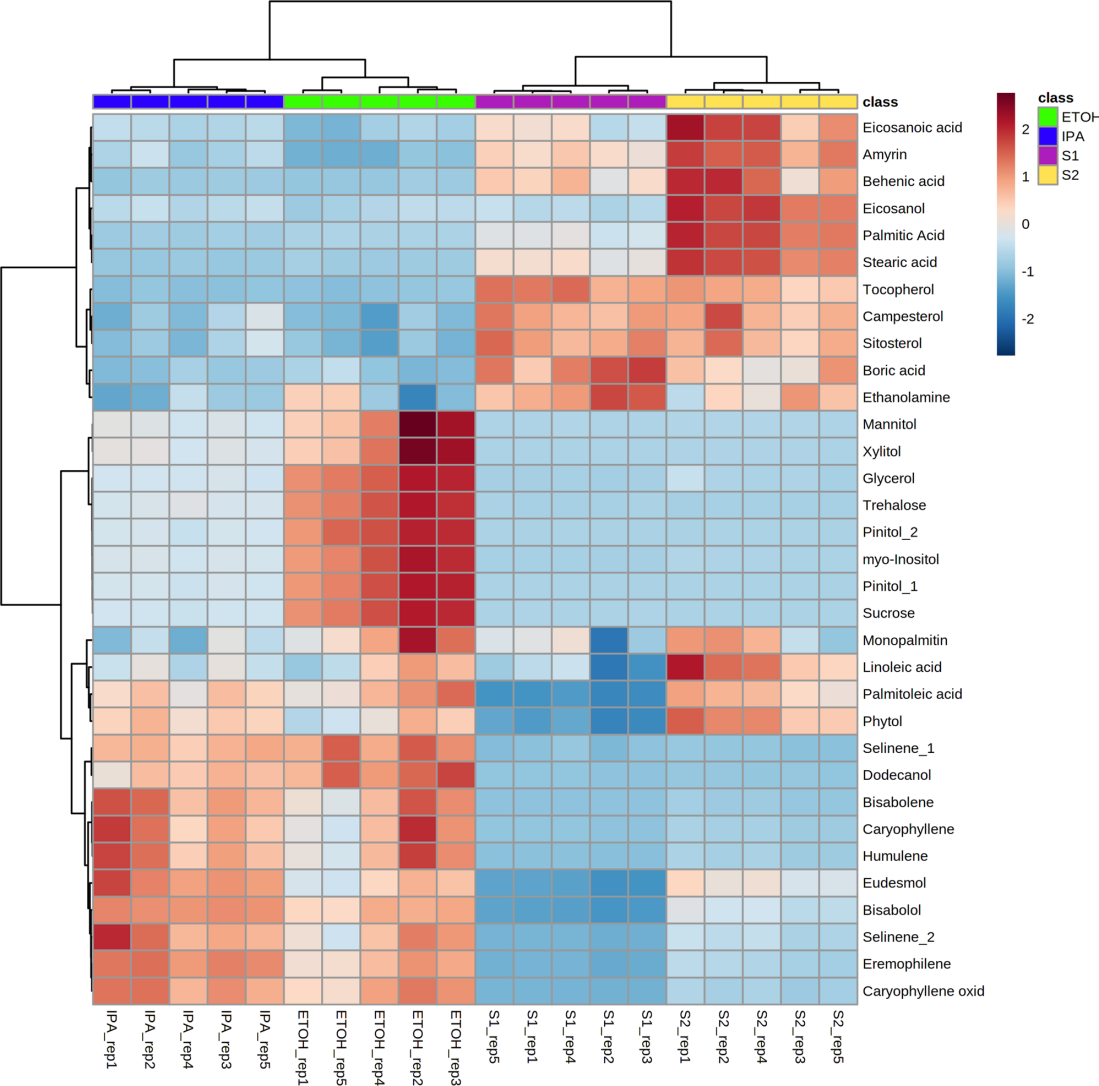
Heatmap of the compounds detected and annotated by GC–MS and significantly different in abundance across the 4 extracts (p < 0.05 after Tukey pos-hoc testing for multiple comparisons). Green = ethanol extract; Blue = isopropanol extract; Purple and Yellow = super critical CO_2_ fractions S1 and S2, respectively.

Principal Component Analysis (PCA) was performed for the compounds detected by GC–MS demonstrating that there is significant variation in the overall chemical profiles between samples based on extraction method (Fig. 1). Of the 41 annotated compounds detected by GC–MS, 33 were significantly different between at least 2 of the extracts (Table S1, Fig. 2; p < 0.05 after Tukey post-hoc testing for multiple comparison). These compounds include multiple long chain fatty acids, polyols and carbohydrates, and sesqui-, tri-, and diterpenoids (Table S2). Compound annotations were determined based on searching against spectral databases and not comparison to authentic standards. Thus, when structural isomers could not be distinguished annotations are denoted with a number (Fig. 2, e.g. pinitol). The data reveal significant differences that could result in variation of therapeutic potential of the product (Fig. 2). For example, sitosterol was significantly enriched in the S1 and S2 fractions as compared to IPA and EtOH. This compound has multiple known health benefits and is used as a potential prevention and therapy for treatment of cancer and as an anticholesteremic drug^32^. Bisabolol was significantly enriched in the IPA and EtOH extracts as compared to S1 and S2. This compound has known anti-inflammatory and anti-microbial properties^33^. Palmitoleic acid is a carboxylesterase inhibitor that has anti-inflammatory effects^34^ that was enriched in the IPA, EtOH, and S2 extracts. Campesterol was enriched in the S1 and S2 extracts compared to IPA and EtOH. Plant sterols such as campesterol are cholesterol-lowering compounds^35^ that may act in cancer prevention^36^.

**Figure 3:**
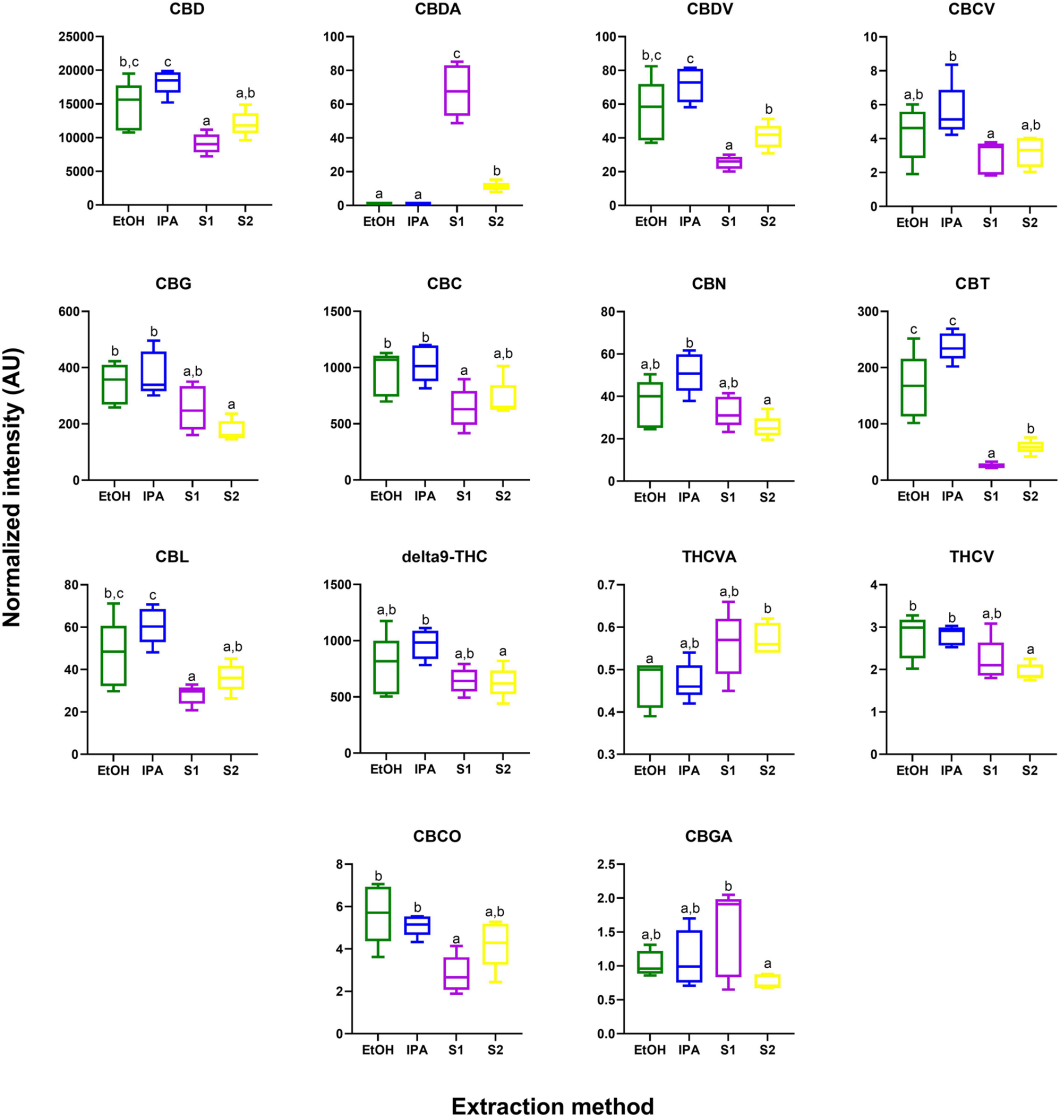
Phytocannabinoids significantly different in abundance by extraction method (p < 0.05 after Tukey pos-hoc testing for multiple comparisons). Box plots indicate relative abundance of each compounds between extractions. Green = ethanol extract; Blue = isopropanol extract; Purple and Yellow = super critical CO_2_ fractions S1 and S2, respectively.

While cannabinoids were detected by GC–MS (Table S1), the unregulated decarboxylation that occurs in the ionization source complicates interpretation; thus, a complementary analysis was performed using a targeted UPLC-MS/MS assay. From this analysis, 14 of the 15 detected phytocannabinoids were significantly different across the extracts (Fig. 3, Table S3; p < 0.05 after Tukey post-hoc testing for multiple comparison).

In general, there is a trend of higher abundance of phytocannabinoids (including CBD and Δ^9^THC) in the EtOH and IPA extracts compared to the supercritical CO_2_ fractions (S1 and S2). A notable break in this trend was observed for CBDA which was observed to be in highest abundances in the supercritical CO_2_ S1 extract. This result could reflect incomplete decarboxylation of CBDA to CBD which was performed by heating the dried plant material prior to extraction with supercritical CO_2_. This is in contrast to the process used when extracting by EtOH and IPA, where decarboxylation was performed in the liquid phase post extraction.

Interestingly, in addition to differences in the major phytocannabionds, significant differences were observed for multiple under-researched minor phytocannabinoids. For example, CBC was observed to be significantly more abundant in the IPA and EtOH fractions as compared to S1. CBC acts as a CB2 receptor agonist that has anti-nociceptive and anti-inflammatory effects^37,38^. CBC has been implicated as a potential anti-depressant in previous in vivo studies^46^. Furthermore, CBC can act as an agonist for TRPA1 channels, as demonstrated in an ex vivo study using isolated nerves from rats^47^. It has also been implicated as an analgesic for pain localized on efferent neural pathways^39^.

The largest differences in abundance (higher in EtOH and IPA as compared to both S1 and S2) were observed for CBT, a minor phytocannabinoid found in cannabis varieties at trace levels. Intriguingly, this compound is also found in one species of rhododendron, the specific type used in traditional Chinese medicine to treat bronchitis and other respiratory ailments^48^. In one of the only in vivo studies to date, CBT was found to decrease the intraocular pressure in rabbit, suggesting CBT as a potential therapeutic for glacuoma^49^.

**Figure 4:**
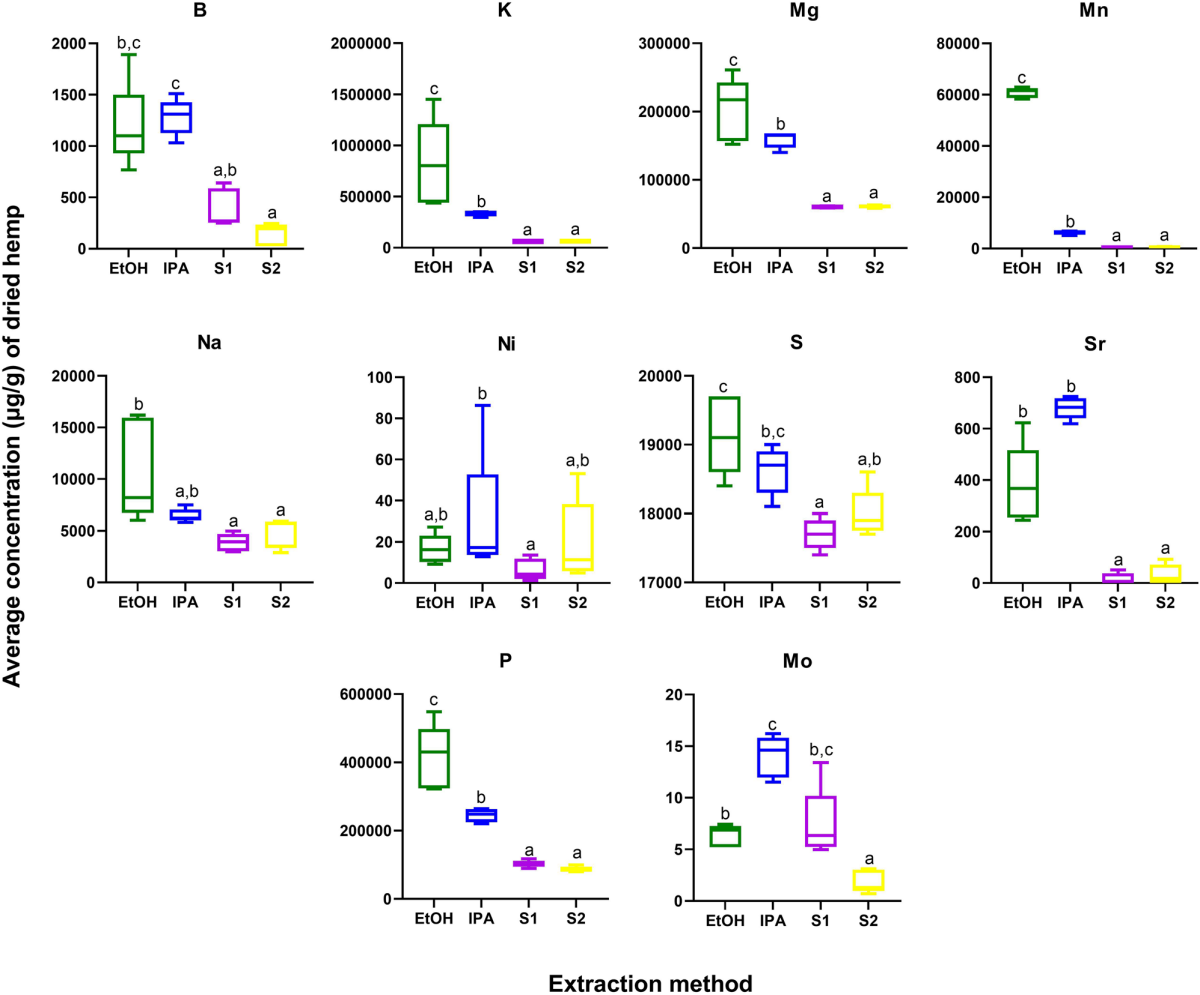
Elements significantly different in concentration by extraction method (p < 0.05 after Tukey pos-hoc testing for multiple comparisons). Green = ethanol extract; Blue = isopropanol extract; Purple and Yellow = super critical CO_2_ fractions S1 and S2, respectively.

Cannabis plants have a wide root system which can facilitate efficient uptake of elements from the soil. Cannabis products, in particular seed extracts, have been shown to be a good source of both micro- and macro-elements including P, K, Mg, Ca, Fe, Zn, Cu, and Mn. In addition to the absorption of beneficial nutrients, cannabis plants can also be exploited for the intentional phytoremediation of toxic heavy metals from soil^40^. Here, we performed ionomics profiling of 24 elements including nutrients, minerals, and toxic heavy metals. Ten elements (B, K, Mg, Mn, Na, Ni, S, Sr, P, and Mo) were significantly different across the 4 extracts, and all of these were higher in abundance in EtOH and/or IPA as compared to the supercritical CO_2_ fractions (Fig. 4, Table S4). Importantly, none of the toxic heavy metals (Cd, Pb, and As) were significantly impacted by extraction method and none were detected above regulatory levels set by the state of California.

Taken together, the results presented here highlight the importance of the extraction process in the overall chemical profile of the resulting extract which will determine the potential bioactivity of the product. This is particularly important in the generation of so called “full spectrum”, “whole plant”, and/or “broad spectrum” extracts which are becoming increasingly popular as consumer products but are not supported by research related to therapeutic efficacy. While comprehensive detection and characterization of all potential molecular components in a complex matrix remains a grand challenge^41^, the approaches used in this study represent a relevant snapshot of the major chemical components of these extracts and lay the groundwork for future studies to explore the influence of extraction method on therapeutic potential of full spectrum products. Importantly, these results demonstrate the need for improved transparency and regulation regarding how full spectrum cannabis products are labeled to protect consumers and enable accurate interpretation and comparison of multi-component products.

## Materials and methods

The use of plants in the present study complies with international, national, and institutional guidelines. Cannabis material was grown and extracted by a commercial supplier certified by the state of Colorado Department of Agriculture (Industrial Hemp Registration #102,273). All plant material (mixture of inflorescence, stem, and fan leaves) used in the three extraction methods was from a single cultivar and the same production lot. Five unique sampling replicates were used for each extraction method. The cannabis plants were air dried under ambient conditions. The dried plants (< 5% water (w/w)) were stored in the dark at 25 °C prior to homogenization and extraction. Seeds, stems, and larger petioles were removed from the dried flower using a combine prior to homogenization. Material was homogenized with a hammer mill until a particle size of approximately 2–3 mm was accomplished.

### Alcohol extraction

For each replicate, 7 g of dried plant material was weighed into a 100 mL media bottle. The bottle was wrapped and fully covered with aluminum foil to limit UV light exposure to the plant material inside. 75 mL of the appropriate extraction solvent (ethanol (EtOH; food grade), or isopropyl alcohol (IPA; food grade) was added to each flask followed by shaking (via flask shaker) at 150 rpm for 30 min at room temperature. The miscella was decanted into a tared 250 mL round bottom flask wrapped in aluminum foil. Remaining plant material was subjected to vacuum filtration (25 µm pore size). The plant material was rinsed with several 5 mL aliquots of solvent until the filtered liquid turned visibly clear. The filtrate was combined with the decanted liquid in the round bottom flask and subjected to reduced pressure evaporation at 50 °C using a rotary evaporator. When the extracts were largely evaporated yet moderately viscous (as determined by visual inspection) they were transferred to 40 mL amber-colored scintillation vials incubated at 135 °C for 40 min for decarboxylation. After cooling to room temperature samples were diluted with ethanol and stored at − 20 °C until analysis.

### Super critical fluid extraction (SFE-CO_2_)

Dried plant material was heated in a 122 °C conventional convection oven for 20 min. 1.5 kg of milled, homogenized plant biomass was placed in a supercritical CO_2_ extractor (Hightech Dual P-12, Biddeford, ME). The supercritical fluid was heated to 53 °C and subjected to a pressure of 250 bar. The first separator (S1) was set to a pressure of 170 bar while the second separator (S2) was set to a pressure of 70 bar; both separators were set to 53 °C. The third separator pressure depended on the settings of the first two and was used as a trap for unwanted carryover substances (water) before returning back to the CO_2_ tank (food grade). The flow rate was set to 45 kg CO_2_/hour, and the extraction cycle lasted 60 min. After the 60-min cycle, two fractions were obtained through sequential depressurization from 170 Bar (S1) to system pressure (S2, 70 bar). The S1 and S2 separators were emptied of their contents and placed in several 40 mL amber scintillation vials. Extracts were homogenized, diluted with isopropyl alcohol, and stored at − 20 °C until analysis.

Extract weights from all protocols ranged from 0.31–0.80 g and were diluted to 0.05 (+ /- 0.02) g/mL in either ethanol or isopropyl alcohol prior to sample preparation as described below.

### Materials for analysis

Pyridine, methoxyamine hydrochloride, N-methyl-N-trimethylsilyltrifluoroacetamide with 1% trimethylchlorosilane (MSTFA + 1% TMCS), water (LC–MS grade), methanol (LC–MS grade), formic acid (Pierce LC–MS grade), and acetonitrile (LC–MS grade) were purchased from Thermo Fisher Scientific (Waltham, MA). 20 phytocannabinoid standards including CBD, CBC, CBCA, CBCO, CBCV, CBDA, CBDV, CBDVA, CBG, CBGA, CBL, CBLA, CBN, CBNA, CBT, Δ9-THC, Δ8-THC, Δ9-THCA, Δ9-THCV, and Δ9-THCVA were purchased from Cerilliant (Round Rock, TX). Single element standards for ICP-MS analysis including Al, As, B, Ba, Ca, Cd, Co, Cr, Cu, Fe, In, Ir, K, Li, Mg, Mn, Mo, Na, Ni, P, Pb, Rh, S, Sr, V, W, and Zn were purchased from Inorganic Ventures (Christiansburg, VA).

*GC–MS Analysis.* Non-targeted GC–MS profiling was performed using established methods as previously described^42^. Briefly, 100 μL of each extract was dried under nitrogen. The dried sample was re-suspended in 90 µL of 4:4:1 MeOH:ACN:H_2_O, vortexed for 1 h at 4 °C, and then centrifuged at 12700xg for 15 min at 4 °C. Ten microliters of the supernatant was transferred to a new vial and dried under nitrogen. The dried sample was re-suspended in 50 µL of pyridine containing 25 mg/mL of methoxyamine hydrochloride, incubated at 60˚C for 1 h, sonicated for 10 min, and incubated for an additional 1 h at 60 °C. Next, 50 µL of MSTFA + 1% TMCS was added, and samples were incubated at 60 °C for 45 min, briefly centrifuged, cooled to room temperature, and 80 µL of the supernatant was transferred to a 150 µL glass insert in a GC–MS autosampler vial. Metabolites were detected using a Trace 1310 GC coupled to a Thermo ISQ mass spectrometer (Thermo Scientific). Derivatized samples (1 µL) were injected in a 1:10 split ratio. Separation occurred using a 30 m TG-5MS column (Thermo Scientific, 0.25 mm i.d., 0.25 mm film thickness) with a 1.2 mL/min helium gas flow rate, and the program consisted of 80ºC for 30 s, a ramp of 15 °C per min to 330 °C, and an 8 min hold. Masses between 50–650 m/z were scanned at 5 scans/sec after electron impact ionization.

### UPLC-MS/MS analysis

Ten microliters of each extract were diluted in 990 µL of LC–MS grade methanol. Subsequently, 10 µL of the methanol solution was diluted again to a total volume of 200 µL of LC–MS grade MeOH. Five microliters of the final diluted extract were injected onto an LX50 UHPLC system equipped with a LX50 solvent delivery pump (20 µL sample loop, partial loop injection mode; PerkinElmer, Shelton, CT, USA). An ACQUITY UPLC HSS T3 column (1 × 100 mm, 1.8 µM; Waters Corporation, Milford, MA, USA) was used for chromatographic separation. The column was maintained at 50 °C, mobile phase A consisted of LC–MS grade water with 0.1% formic acid and mobile phase B was 100% acetonitrile. Elution gradient was initially at 59% B for 11.5 min, which was increased to 99% B at 16.50 min, then decreased to 59% B at 21.5 min. The column was re-equilibrated for 4 min for a total run time of 25.50 min. The flow rate was set to 200 µL/min. Detection was performed on a PerkinElmer QSight 220 triple quadrupole mass spectrometer (MS) with an electrospray ionization source operated in selected reaction monitoring (SRM) switching from negative and positive mode ionization. SRM transitions for each compound were optimized through analysis of authentic standards (Table S5). The MS had a drying gas 120 (arbitrary units), a hot-surface induced desolvation (HSID) temperature of 250 °C, electrospray voltage was kept at − 4500 eV or 4500 eV, and a nebulizer gas flow at 350 (arbitrary units). The MS acquisition was scheduled by retention time with 1.5 min windows.

### GC–MS data analysis

The GC–MS dataset was processed using the R statistics software as described previously^43^. Briefly, the following processing steps were performed: (1) XCMS was used to defined a matrix of molecular features^44^, (2) data in each sample were normalized to total ion current, (3) RAMClustR package for R clustered co-varying and co-eluting features into spectra^45^, (4) RAMSearch software^46^ (https://osf.io/x8bw5/) allowed annotation by searching spectra against internal and external databases. Databases used for annotations included in-house libraries, golm (http://gmd.mpimp-golm.mpg.de/), NISTv14 (http://www.nist.gov), and MassBank (http://www.massbank.jp). Principal component analysis (PCA) was performed using unit variance scaling in SIMCA v15.0.2 (Umetrics, Umea, Sweden). The MetaboAnalyst (http://www.metaboanalyst.ca/) was used to perform hierarchical clustering using the hclust function in the R package stat (euclidean distance measure, ward clustering algorithm) and to generate heatmap visualization^47^. Univariate statistical analysis was performed in Prism (Version 8.2.1, GraphPad). Data was log transformed and then analyzed using a one way ANOVA with a Tukey’s multiple comparisons test.

### UPLC-MS/MS data analysis

Data processing was performed using Simplicity 3Q software (Version 1.5, PerkinElmer). Peak retention times corresponding to SRM transitions for each phytocannabinoid were validated against authentic standards. Peak areas were integrated, normalized to total ion current, and exported for statistical analysis performed in GraphPad Prism. Data was log transformed and then analyzed using a one way ANOVA with a Tukey’s multiple comparisons test.

### ICP-MS sample preparation

ICP-MS sample preparation and analysis was performed based on modified methods previously described^48^. Briefly, 100 μL of each extract was dried under nitrogen and then digested with 429 µL of redistilled concentrated nitric acid (HNO_3_) spiked with indium (internal standard, In, 349.65 ppb). Samples reacted at room temperature overnight and were subsequently placed in a shaking incubator at 70 °C for 2 h. The samples were allowed to cool to room temperature, followed by the addition of 200 µL of ultra-trace grade H_2_O_2_. Samples were left to react at room temperature for 2 h and then placed in a shaking incubator at 70 °C for 1 h. After cooling again to room temperature, samples were diluted with Type 1 water (MilliQ 18.2 MΩ) to a final volume of 15 mL, resulting in a sample matrix consisting of 10 ppb of In and 2.5% HNO_3_. Additionally, 1 mL of each sample was combined to generate a pooled QC sample.

### ICP-MS analysis

Elemental concentrations were measured using an NexION 350D mass spectrometer (PerkinElmer, Branford, CT) connected to a PFA-ST (Elemental Scientific, Omaha, Nebraska) nebulizer and a Peltier controlled (PC3x, Elemental Scientific) quartz cyclonic spray chamber (Elemental Scientific) set at 4 °C. Samples were introduced using prepFAST SC-2 (Elemental Scientific) autosampler. Analysis was performed in a randomized order utilizing standard mode and dynamic reaction mode using oxygen or ammonia as the reactive gas with analysis of the pooled QC after every 9 samples. Before analysis, a daily performance check was run to ensure instrument performance. A calibration curve was prepared by serial dilution of single element standard stock solutions. These were matrix matched to the samples, 2.5% HNO_3_ and 10 ppb of In. For correction of instrument drift internal standard solution consisting of ^6^Li, Rh and Ir was added to each sample via the autosampler.

### ICP-MS data analysis and statistics

Data was processed using Excel (Microsoft Office). Each element was subjected to internal standard corrections and subsequently drift corrected^49^. Corrections were chosen based on minimizing the coefficient of variance for the QC samples. Limits of detection (LOD) and limits of quantification (LOQ) were calculated as 3 times or 10 times the standard deviation of the blank divided by the slope of the calibration curve respectively^50,51^. Final concentrations were determined in units of µg/g based on initial starting mass of each extract. Measured calculations below the LOQ were assigned to LOQ/2^52^. Data was analyzed using one way ANOVA with a Tukey’s multiple comparisons test in GraphPad Prism.

## Supplementary Information

Supplementary Information 1.

Supplementary Information 2.

## Data Availability

Data matrices representing integrated peak areas for GC–MS and LC–MS/MS analysis, and absolute quantitation (including LOD and LOQ values) for ICP-MS analysis are available as supplemental data.
