# Supplementary material for: The impact of extraction protocol on the chemical profile of cannabis extracts from a single cultivar
Source: Sci Rep. 2021 Nov 8;11:21801. doi: 10.1038/s41598-021-01378-0 (PMC8575894; doi:10.1038/s41598-021-01378-0)
Supplement: Supplementary file 1 — Supplementary Information 1. [file 41598_2021_1378_MOESM1_ESM.docx]

The impact of extraction protocol on the chemical profile of cannabis extracts from a single cultivar

*Janina K. Bowen*^†^*, Jacqueline M. Chaparro*^†^*, Alexander McCorkle*^‡^*, Edward Palumbo*^‡^*, and Jessica E. Prenni*^†^*^*^*

^†^ Department of Horticulture and Landscape Architecture, Colorado State University, Fort Collins, CO

^‡^Charlotte’s Web Inc.,1600 Pearl St. Boulder CO, 80302

*corresponding author: [jprenni@colostate.edu](mailto:jprenni@colostate.edu); 970-491-3664

**Supplementary Information**

**Table S1.** Results from one way ANOVA analysis (log transformed data) of 40 annotated compounds detected by GC-MS from 4 different extracts. ETOH = ethanol, IPA = isopropyl alcohol, S1 and S2 = super critical CO_2_ fractions. Reported p-values are adjusted for multiple comparisons using a Tukey post-hoc test. Values in red indicate statistical significance.

|  | ETOH vs. IPA | ETOH vs. S1 | ETOH vs. S2 | IPA vs. S1 | IPA vs. S2 | S1 vs. S2 |
| --- | --- | --- | --- | --- | --- | --- |
| Selinene_1 | 0.3570 | <0.0001 | <0.0001 | <0.0001 | <0.0001 | 0.3055 |
| Bisabolol | 0.0713 | <0.0001 | <0.0001 | <0.0001 | <0.0001 | <0.0001 |
| Amyrin | 0.0036 | <0.0001 | <0.0001 | <0.0001 | <0.0001 | <0.0001 |
| Bisabolene | 0.4933 | <0.0001 | <0.0001 | <0.0001 | <0.0001 | 0.0109 |
| Eudesmol | 0.0050 | <0.0001 | 0.7787 | <0.0001 | 0.0007 | <0.0001 |
| THC_1 | 0.0097 | <0.0001 | <0.0001 | <0.0001 | <0.0001 | <0.0001 |
| THC_2 | 0.3341 | 0.0203 | 0.2951 | 0.4197 | 0.9997 | 0.4671 |
| Monopalmitin | 0.0466 | 0.0529 | 0.7109 | 0.999 | 0.2977 | 0.3272 |
| Eremophilene | 0.0192 | <0.0001 | <0.0001 | <0.0001 | <0.0001 | <0.0001 |
| Apigenin | 0.9957 | 0.1710 | 0.2368 | 0.1159 | 0.1642 | 0.9970 |
| Behenic acid | 0.9789 | <0.0001 | <0.0001 | <0.0001 | <0.0001 | 0.0289 |
| Boric acid | >0.9999 | <0.0001 | 0.0005 | <0.0001 | 0.0006 | 0.5240 |
| Campesterol | 0.4140 | <0.0001 | <0.0001 | <0.0001 | <0.0001 | >0.9999 |
| Cannabidiol_1 | 0.9685 | 0.0050 | 0.0116 | 0.0020 | 0.0046 | 0.9761 |
| Cannabinol_2 | 0.0053 | 0.1675 | 0.4221 | 0.3139 | 0.0002 | 0.0083 |
| Cannabinol_3 | 0.0912 | 0.4107 | <0.0001 | 0.7797 | <0.0001 | <0.0001 |
| Caryophyllene | 0.6038 | <0.0001 | <0.0001 | <0.0001 | <0.0001 | 0.0001 |
| Caryophyllene oxide | 0.4432 | <0.0001 | <0.0001 | <0.0001 | <0.0001 | <0.0001 |
| Mannitol | <0.0001 | <0.0001 | <0.0001 | <0.0001 | <0.0001 | 0.6558 |
| Dodecanol | 0.0175 | <0.0001 | <0.0001 | <0.0001 | <0.0001 | 0.0009 |
| Pinitol_1 | <0.0001 | <0.0001 | <0.0001 | <0.0001 | <0.0001 | 0.9154 |
| Eicosanol | 0.3554 | 0.7015 | <0.0001 | 0.9266 | <0.0001 | <0.0001 |
| Eicosanoic acid | 0.3094 | 0.0039 | <0.0001 | 0.1318 | <0.0001 | 0.0003 |
| Ethanolamine | 0.8344 | 0.0065 | 0.1832 | 0.0012 | 0.0398 | 0.3354 |
| Glycerol | <0.0001 | <0.0001 | <0.0001 | <0.0001 | 0.0001 | 0.0215 |
| Glycerol monostearate | 0.3149 | 0.9355 | 0.9661 | 0.1234 | 0.1521 | 0.9993 |
| Palmitoleic acid | 0.7894 | <0.0001 | 0.9905 | <0.0001 | 0.9200 | <0.0001 |
| Humulene | 0.5378 | <0.0001 | <0.0001 | <0.0001 | <0.0001 | <0.0001 |
| myo-Inositol | <0.0001 | <0.0001 | <0.0001 | <0.0001 | <0.0001 | 0.0018 |
| Linoleic acid | 0.7882 | 0.0341 | 0.2071 | 0.1869 | 0.0386 | 0.0005 |
| Pinitol_2 | <0.0001 | <0.0001 | <0.0001 | <0.0001 | <0.0001 | 0.9493 |
| Palmitic Acid | 0.0003 | <0.0001 | <0.0001 | <0.0001 | <0.0001 | <0.0001 |
| Phytol | 0.4923 | <0.0001 | 0.0357 | <0.0001 | 0.4105 | <0.0001 |
| Selinene_2 | 0.2818 | <0.0001 | 0.0015 | <0.0001 | <0.0001 | <0.0001 |
| Sitosterol | 0.2813 | <0.0001 | <0.0001 | <0.0001 | <0.0001 | 0.8063 |
| Stearic acid | 0.0117 | <0.0001 | <0.0001 | <0.0001 | <0.0001 | <0.0001 |
| Sucrose | <0.0001 | <0.0001 | <0.0001 | >0.9999 | <0.0001 | <0.0001 |
| Tocopherol | 0.9115 | <0.0001 | <0.0001 | <0.0001 | <0.0001 | 0.0525 |
| Trehalose | <0.0001 | <0.0001 | <0.0001 | <0.0001 | <0.0001 | 0.1309 |
| Xylitol | <0.0001 | <0.0001 | <0.0001 | <0.0001 | <0.0001 | 0.2945 |

**Table S2.** Chemical class of annotated compounds detected by GC-MS that were significant across the 4 extracts (p < 0.05 after Tukey post-hoc testing for multiple comparisons). Chemical class information was determined from compound entries in the Human Metabolome Database (<https://hmdb.ca/>).

| Compound Annotation | Chemical Class |
| --- | --- |
| Selinene_1 | Sesquiterpenoid_1 |
| Bisabolol | Sesquiterpenoid_2 |
| Amyrin | Triterpenoid_1 |
| Bisabolene | Sesquiterpenoid_3 |
| Eudesmol | Sesquiterpenoid_4 |
| Monopalmitin | Glycerolipid |
| Eremophilene | Sesquiterpenoid_5 |
| Behenic acid | Long chain fatty acid_1 |
| Boric acid | Metalloid oxide |
| Campesterol | Ergostane steroid |
| Caryophyllene | Sesquiterpenoid_6 |
| Caryophyllene oxide | Sesquiterpenoid_7 |
| Mannitol | Carbohydrate_1 |
| Dodecanol | Fatty alcohol_1 |
| Pinitol_1 | Polyol_1 |
| Eicosanol | Fatty alcohol_2 |
| Eicosanoic acid | Long chain fatty acid_2 |
| Ethanolamine | Amine |
| Glycerol | Polyol_2 |
| Palmitoleic acid | Long chain fatty acid_3 |
| Humulene | Sesquiterpenoid_8 |
| myo-Inositol | Polyol_3 |
| Linoleic acid | Long chain fatty acid_4 |
| Pinitol_2 | Polyol_4 |
| Palmitic Acid | Long chain fatty acid_5 |
| Phytol | Diterpenoid_1 |
| Selinene_2 | Sesquiterpenoid_9 |
| Sitosterol | Triterpenoid_2 |
| Stearic acid | Long chain fatty acid_6 |
| Sucrose | Carbohydrate_2 |
| Tocopherol | Diterpenoid_2 |
| Trehalose | Carbohydrate_3 |
| Xylitol | Carbohydrate_4 |

**Table S3.** Results from one way ANOVA analysis (log transformed data) of 15 cannabinoids from 4 different extraction methods. ETOH = ethanol, IPA = isopropyl alcohol, S1 and S2 = super critical CO_2_ fractions. Reported p-values are adjusted for multiple comparisons using a Tukey post-hoc test. Values in red indicate statistical significance.

|  | ETOH vs. IPA | ETOH vs. S1 | ETOH vs. S2 | IPA vs. S1 | IPA vs. S2 | S1 vs. S2 |
| --- | --- | --- | --- | --- | --- | --- |
| **CBT** | 0.0513 | <0.0001 | <0.0001 | <0.0001 | <0.0001 | 0.0001 |
| **CBDA** | 0.5935 | <0.0001 | <0.0001 | <0.0001 | <0.0001 | <0.0001 |
| **CBDV** | 0.2157 | 0.0002 | 0.2073 | <0.0001 | 0.0042 | 0.0149 |
| **CBD** | 0.1810 | 0.0040 | 0.4036 | <0.0001 | 0.0085 | 0.0946 |
| **CBL** | 0.2057 | 0.0216 | 0.4251 | 0.0003 | 0.0108 | 0.3443 |
| **CBCO** | 0.9604 | 0.0025 | 0.3014 | 0.0068 | 0.5591 | 0.0925 |
| **CBC** | 0.9182 | 0.0426 | 0.2149 | 0.0120 | 0.0707 | 0.8026 |
| **CBG** | 0.9183 | 0.1927 | 0.0022 | 0.0623 | 0.0006 | 0.1378 |
| **CBCV** | 0.5327 | 0.3834 | 0.6536 | 0.0369 | 0.0907 | 0.9636 |
| **CBN** | 0.1287 | 0.9218 | 0.1502 | 0.0402 | 0.0015 | 0.3906 |
| **delt9THC** | 0.3151 | 0.7570 | 0.6305 | 0.0587 | 0.0386 | 0.9961 |
| **CBGA** | 0.9989 | 0.4962 | 0.4956 | 0.5796 | 0.4168 | 0.0488 |
| **CBNA** | 0.9978 | 0.5832 | 0.9840 | 0.4778 | 0.9980 | 0.3835 |
| **THCVA** | 0.9985 | 0.0909 | 0.0387 | 0.1210 | 0.0526 | 0.9682 |
| **THCV** | 0.9950 | 0.1399 | 0.0126 | 0.0917 | 0.0078 | 0.6001 |

**Table S4.** Results from one way ANOVA analysis (log transformed data) of 24 elements from 4 different extracts. ETOH = ethanol, IPA = isopropyl alcohol, S1 and S2 = super critical CO_2_ fractions. Reported p-values are adjusted for multiple comparisons using a Tukey post-hoc test. Values in red indicate statistical significance.

|  | ETOH vs. IPA | ETOH vs. S1 | ETOH vs. S2 | IPA vs. S1 | IPA vs. S2 | S1 vs. S2 |
| --- | --- | --- | --- | --- | --- | --- |
| **Al** | 0.9995 | 0.969 | 0.9329 | 0.986 | 0.9616 | 0.9989 |
| **As** | 0.9995 | 0.969 | 0.9329 | 0.986 | 0.9616 | 0.9989 |
| **B** | 0.9939 | 0.0662 | 0.0001 | 0.0406 | <0.0001 | 0.0248 |
| **Ba** | 0.9941 | 0.9914 | 0.949 | >0.9999 | 0.9917 | 0.9944 |
| **Ca** | 0.9031 | 0.1964 | 0.2458 | 0.0588 | 0.0767 | 0.9989 |
| **Cd** | >0.9999 | 0.1245 | 0.0567 | 0.1305 | 0.0597 | 0.9726 |
| **Co** | 0.9586 | 0.2815 | 0.3765 | 0.1255 | 0.1783 | 0.9968 |
| **Cr** | 0.8706 | 0.9999 | 0.3466 | 0.8979 | 0.7748 | 0.3797 |
| **Cu** | 0.9489 | 0.8874 | 0.7421 | 0.6015 | 0.9623 | 0.3376 |
| **Fe** | 0.9374 | 0.4744 | 0.9993 | 0.2107 | 0.8959 | 0.5446 |
| **K** | 0.0013 | <0.0001 | <0.0001 | <0.0001 | <0.0001 | >0.9999 |
| **Li** | 0.1393 | 0.1393 | 0.1111 | >0.9999 | 0.9992 | 0.9992 |
| **Mg** | 0.0469 | <0.0001 | <0.0001 | <0.0001 | <0.0001 | 0.9984 |
| **Mn** | 0.0002 | <0.0001 | <0.0001 | <0.0001 | <0.0001 | 0.9986 |
| **Mo** | 0.0238 | 0.9765 | 0.0002 | 0.0527 | <0.0001 | <0.0001 |
| **Na** | 0.1617 | 0.0007 | 0.0061 | 0.0608 | 0.3553 | 0.7191 |
| **Ni** | 0.8985 | 0.1272 | 0.9942 | 0.0349 | 0.7823 | 0.1943 |
| **P** | 0.0001 | <0.0001 | <0.0001 | <0.0001 | <0.0001 | 0.2402 |
| **Pb** | 0.8182 | 0.432 | 0.921 | 0.1121 | 0.9947 | 0.1708 |
| **S** | 0.2089 | 0.0001 | 0.0015 | 0.0076 | 0.0894 | 0.6007 |
| **Sr** | 0.9621 | 0.0039 | 0.0239 | 0.0015 | 0.0091 | 0.8101 |
| **V** | 0.6679 | 0.1258 | 0.9287 | 0.014 | 0.3313 | 0.3301 |
| **W** | 0.9938 | 0.9862 | 0.9996 | 0.9998 | 0.9839 | 0.971 |
| **Zn** | 0.849 | 0.4872 | 0.8874 | 0.149 | 0.9997 | 0.1731 |

**Table S5.** Selected Reaction Monitoring (SRM) transitions for qualitative targeted phytocannabinoid analysis.

|  | RT (min) | Primary Transition | Confirmatory Transition | Polarity | CE (eV) | Dwell Time (ms) |
| --- | --- | --- | --- | --- | --- | --- |
| **CBT** | 14.65 | 315.1 -> 193.1 | 315.1 -> 122.9 | + | -34 | 138 |
| **CBDA** | 3.70 | 357 -> 107 | 357 -> 245.1 | - | 56 | 150 |
| **CBDV** | 2.42 | 287.1 -> 164.9 | 287.1 -> 122.8 | + | -46 | 150 |
| **CBDVA** | 2.11 | 329 -> 107 | 329 -> 217 | - | 37 | 150 |
| **CBD** | 4.66 | 315.1 -> 193 | 315.1 -> 122.9 | + | -31 | 115 |
| **CBL** | 11.16 | 315.1 -> 81.1 | 315.1 -> 165.1 | + | -37 | 250 |
| **CBLA** | 13.67 | 357 -> 191 | 357 -> 216.9 | - | 56 | 138 |
| **CBCO** | 2.98 | 257 -> 135 | 257 -> 146.9 | - | 34 | 150 |
| **CBC** | 12.55 | 315.1 ->193.1 | 315.1 ->122.8 | + | -33 | 250 |
| **CBCA** | 13.87 | 357 -> 136 | 357 -> 147.9 | - | 42 | 138 |
| **CBG** | 4.45 | 317.1 -> 122.9 | 317.1 -> 193 | + | -51 | 115 |
| **CBCV** | 5.72 | 287 -> 122.9 | 287 -> 164.9 | + | -52 | 250 |
| **CBN** | 7.09 | 309 -> 279 | 309 -> 107.9 | - | 46 | 300 |
| **delta9THC** | 9.16 | 315.1 -> 192.9 | 315.1 -> 122.9 | + | -36 | 342 |
| **delat9THCA** | 12.45 | 357 -> 245 | 357 -> 191 | - | 38 | 250 |
| **delta8THC** | 9.76 | 315.1 -> 193 | 315.1 -> 122.8 | + | -62 | 360 |
| **CBGA** | 4.18 | 361.1 -> 343.1 | 343.1 -> 134.9 | + | -20 | 115 |
| **CBNA** | 9.11 | 353 -> 279 | 353 -> 221.9 | - | 49 | 342 |
| **THCVA** | 5.69 | 329 -> 217.1 | 329 -> 163 | - | 41 | 250 |
| **THCV** | 4.23 | 287 -> 165.1 | 387 -> 122.9 | + | -33 | 115 |
